# Supplementary material for: The State of the Art and Prospects for Osteoimmunomodulatory Biomaterials
Source: Materials (Basel). 2021 Mar 11;14(6):1357. doi: 10.3390/ma14061357 (PMC7999637; doi:10.3390/ma14061357)
Supplement: Supplementary file 1 [file materials-14-01357-s001.pdf]

Supplementary Materials

The State of the Art and Prospects for OsteoimmunomodulatoryBiomaterials

Andreea-Mariana Negrescu, Anisoara Cimpean *

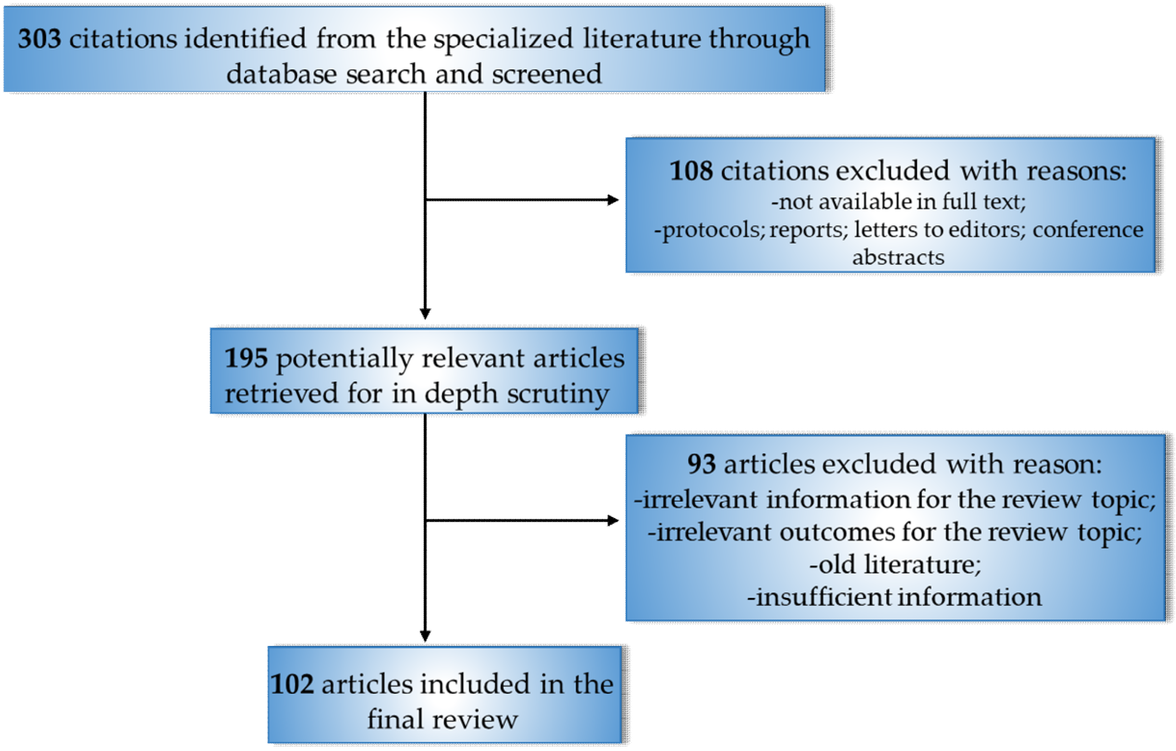

Figure S1. Flowchart of the article selection process.
